# Third Generation Genome Sequencing of the Endobacterium *Corynebacterium kroppenstedtii* subsp. *demodicis* Reveals Details of Its Microbe‐Host‐Interaction With the Most Complex Human Commensal, *Demodex folliculorum*

**DOI:** 10.1111/1758-2229.70374

**Published:** 2026-06-08

**Authors:** T. Steegmüller, S. Walch, S. Gschwendtner, A. Klingl, L. E. French, M. Flaig, B. M. Clanner‐Engelshofen

**Affiliations:** ^1^ Department of Dermatology and Allergy University Hospital, LMU Munich Munich Germany; ^2^ Helmholtz Zentrum München, German Research Center for Environmental Health, Research Unit Comparative Microbiome Analysis Neuherberg Germany; ^3^ Plant Development & Electron Microscopy, Biocenter, Ludwig‐Maximilans‐Universität München Planegg‐Martinsried Germany; ^4^ Dr. Philip Frost, Department of Dermatology and Cutaneous Surgery University of Miami Miller School of Medicine Miami Florida USA

**Keywords:** Demodicidae, endosymbiont, mite, parasite, symbiosis

## Abstract

*Demodex* mites inhabit the pilosebaceous unit despite harsh environmental conditions including UV radiation, variable salinity, and cosmetics. Their recently characterized endobacterium may contribute to this resilience. This study aimed to elucidate mechanisms of the microbe–host interaction that help mites withstand environmental stress. The genome of *Corynebacterium kroppenstedtii* subsp. *demodicis* was sequenced using PacBio technology and annotated via MicroScope. Metabolic and symbiotic traits were analyzed using KEGG and compared with the *Demodex folliculorum* secretome from published transcriptome data. The complete 2,456,075 bp genome contains 2034 coding sequences and exhibits reduced variable genes compared to other *Corynebacterium* species. Primary metabolism comprises an almost complete minimal gene set but lacks two tRNA synthetases and genes for phosphatidylethanolamine and NAD^+^ biosynthesis. Carbohydrate pathways are incomplete and fatty acid synthase I is absent. Secondary metabolism includes complete mevalonate and β‐carotene biosynthetic pathways, while the methylerythritol phosphate pathway is missing. UV protection and oxidative stress tolerance are supported by β‐carotene, ClpB, RecN, MsrA, KatA, SodA, and manganese transporter SitB. The secretome contains hydrolases likely aiding mite digestion. These findings provide genomic insights into mite–bacterium symbiosis and follicular adaptation. All functional inferences are based on genomic data and in silico predictions; experimental validation remains to be established.

## Graphical abstract

The symbiotic interaction between *Demodex folliculorum* and *Corynebacterium kroppenstedtii* subsp. *demodicis*. Created by the author based on genomic and functional analyses presented in this work. The tripartite interaction between the human host, the mite *Demodex folliculorum*, and its endosymbiont *Corynebacterium kroppenstedtii* subsp. *demodicis*. The human skin environment provides a lipid‐rich niche and nutrients that sustain the mite, which in turn harbours the intracellular bacterium within its digestive tract. A mutualistic relationship between mite and bacterium is indicated by metabolic complementarity, including bacterial contributions to lipid processing and stress protection mechanisms such as β‐carotene production and oxidative stress defense. At the same time, the system is embedded in a host context characterized by immune pressure, including reactive oxygen and nitrogen species. Upon mite degradation, bacterial components may be released into the surrounding tissue, where they can interact with the host immune system and contribute to inflammatory responses associated with rosacea. The schematic thus integrates symbiotic interactions with host‐associated immunological consequences within a unified model.

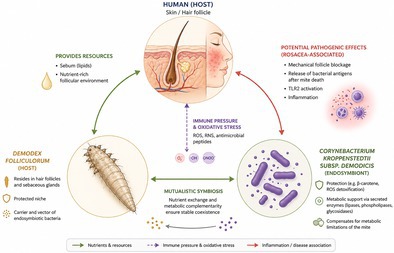

## 1 Introduction

Over 60 years ago, in 1961, Spickett identified acid‐fast bacteria in the digestive tract of *Demodex* mites, leading him to propose the mites as potential vectors of leprosy (Spickett 1961). However, acid‐fastness is not unique to *Mycobacterium leprae* , the causative agent of leprosy, but also occurs in other actinobacteria of the CMNR group (comprising the genera *Corynebacterium*, *Mycobacterium*, *Nocardia* and *Rhodococcus*). Most members of this group have a mycomembrane consisting of mycolic acids that cover the cell wall and lead to high resistance to chemical, physical and antibiotic stress (Burkovski 2018).

One such hostile environment—from a microbial point of view—is human facial skin, characterized by intense heat, extreme exposure to UV‐A and UV‐B radiation, fluctuating salinity, osmotic stress as well as threats from cosmetics and host defense molecules such as dermcidin, cathelicidin and defensins. The resident human skin flora is dominated by gram‐positive bacteria, whose thick peptidoglycan layer aids in environmental stress resistance (Byrd et al. 2018). The most complex resident, however, is the *Demodex* mite—typically a commensal, but capable of becoming pathogenic (Foley et al. 2021). *Demodex folliculorum* inhabits vellus hair follicles and is thought to feed on sebum, an emulsion of lysed sebocytes released via holocrine secretion.

To putatively support digestion, *Demodex* mites harbour an endobacterium recently described by our group as *Corynebacterium kroppenstedtii* subsp. *demodicis* (Clanner‐Engelshofen et al. 2020a). It appears closely related to *Corynebacterium kroppenstedtii* but exhibits some distinctive traits. Though horizontally transmitted, the bacterium is essential for the mite, indicating an obligate symbiosis. To characterize this microbe–host relationship in more detail, we obtained the endobacterial genome via next‐generation sequencing, which also revealed insights into the tripartite interaction between human, mite and endobacterium. The obligate nature of this symbiosis implies that *C. kroppenstedtii* subsp. *demodicis* confers fitness advantages to *Demodex*. Based on genomic predictions, proposed benefits to the mite host include: (i) enzymatic support for digestion of host‐derived lipids, glycolipids and sugars via secreted hydrolases; (ii) protection from UV radiation and oxidative stress through β‐carotene and antioxidant enzyme systems (SodA, KatA, mycothiol); and (iii) potential contributions to nitrogen cycling within the mite midgut. All proposed functions are inferred from genomic data and require experimental confirmation.

## 2 Materials and Methods

### 2.1 Chemicals

All chemicals and buffers were purchased from Sigma Aldrich (Steinheim, Germany), unless otherwise noted.

### 2.2 Endobacteria

Endobacteria (strain Df‐Isolat‐1, ID 18‐284, DSM 109755) were acquired from the German Collection of Microorganisms and Cell Cultures GmbH (DSMZ, Braunschweig, Germany) and isolated and cultured as described before (Clanner‐Engelshofen et al. 2020a).

### 2.3 Mites

*Demodex* mites in human sebum and skin scrapings were obtained anonymously from the diagnostics facility of the department of dermatology at the Ludwig‐Maximilian‐University in Munich, Germany. The uses of anonymized patient samples after diagnostic analysis and anonymized, excised excess facial skin from the operating room of our clinic were approved by the local ethics committee (projects 17‐450UE and 18‐671UE, respectively). Skin samples were used to acquire pore plugs via our previously described ‘peel‐off mask method’ (Clanner‐Engelshofen et al. 2020b). All samples were kept humid and used on the day of collection.

### 2.4 DNA Extraction, Genome Sequencing and Assembly

The complete genome of *Corynebacterium kroppenstedtii* subspecies *demodicis* was sequenced using a single‐molecule real‐time (SMRT) cell 1 M v3 on the Sequel system (Pacific Biosciences [PacBio], Menlo Park, CA, USA). DNA was extracted from 4.5 × 10^9^ cells grown in BHI medium using the Genomic‐tip 20/G kit (Qiagen, Hilden, Germany) per the manufacturer's instructions. The library was multiplexed with other libraries to achieve full SMRT cell capacity. Multiplexed microbial libraries were prepared following the protocol of the SMRTbell Express template preparation kit v2.0 (product number 101‐696‐100, v6 [March 2020]; PacBio) and the Barcoded Overhang Adapter Kit 8A (PacBio). Genomic DNA was sheared to 10 kb using g‐TUBEs (Covaris, Woburn, MA, USA) and processed without additional size selection. The library, with an expected genome size of 2.5 Mbp, was loaded onto a SMRT cell at 6 pM. Libraries were immobilized on the SMRT cells (2 h), pre‐extended (2 h), and then sequenced on the Sequel system using v3.0 chemistry with a 10 h movie time. Data were demultiplexed and the genome assembled using the Microbial Assembly Pipeline in SMRT Link v8.0.0.80529 (PacBio), with a seed coverage of 30×. The genome comprised one large contig. Genome completeness and contamination were assessed using CheckM2 v1.0.1 and BUSCO v5.8.0 with the actinomycetota_odb12 lineage dataset via the Galaxy platform (https://usegalaxy.org) to ensure assembly quality for downstream analyses.

### 2.5 Genome Annotation and Bioinformatic Analysis

**FIGURE 1:**
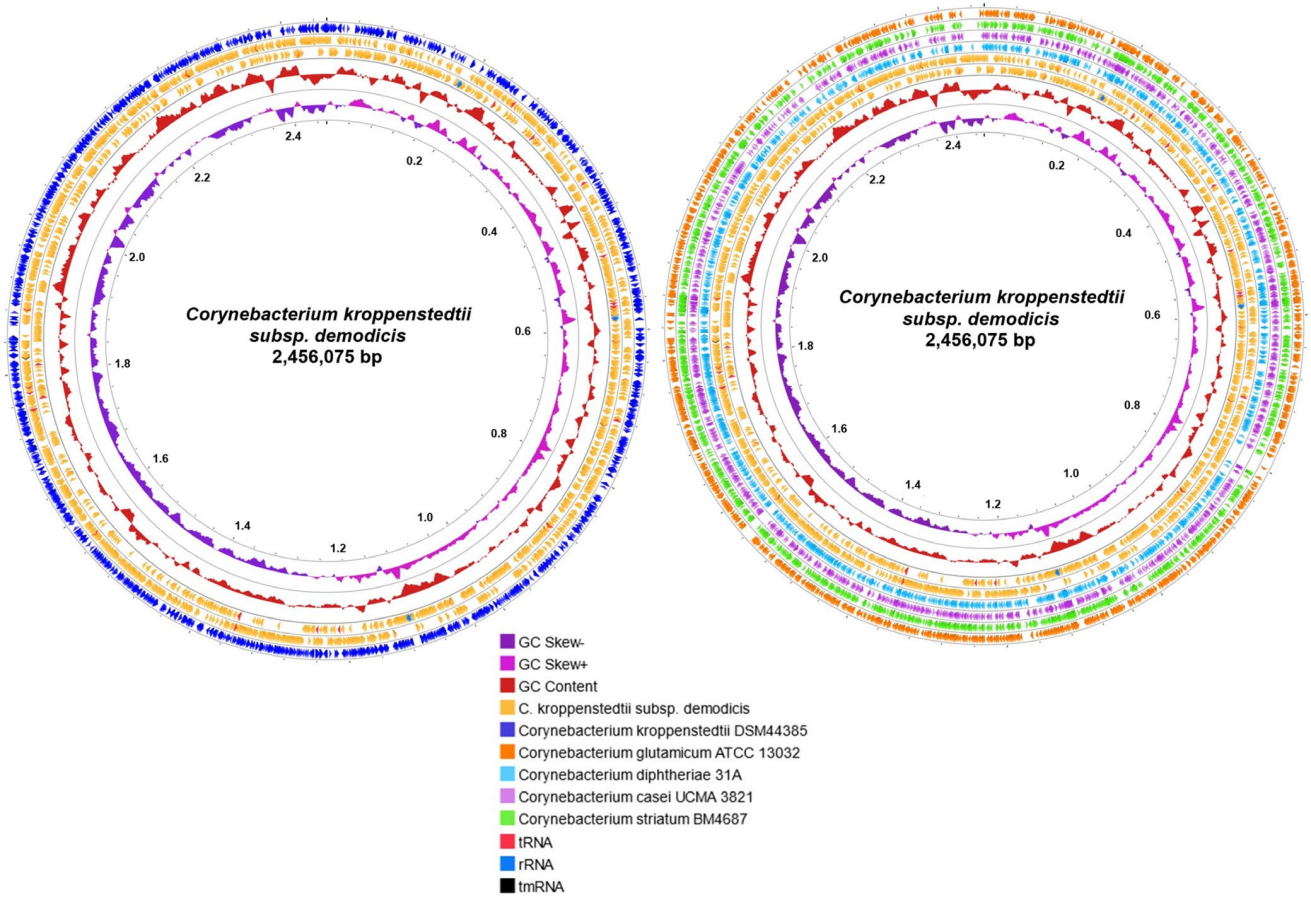
Circular maps of the *C. kroppenstedtii* subsp. *demodicis* genome (yellow) compared to *C. kroppenstedtii* DSM 44385 (dark blue) (left) and *C. glutamicum* (orange), *C. diphtheriae* (light blue), *C. casei* (lilac) and *C. striatum* (green) (right). GC skew is coloured purple (negative) and pink (positive), GC content in red. tRNAs are coloured in red, rRNAs in blue and tmRNA in black (yellow track). Graphs were compiled via Proksee (proksee.ca).

The complete genome of *C. kroppenstedtii* subsp. *demodicis* was uploaded to the MicroScope platform (mage.genoscope.cns.fr) for automatic annotation (Vallenet et al. 2020). Gene prediction and protein characterization were performed using built‐in tools (Figure 1, visualization via Proksee (proksee.ca)). Orthologs among *Corynebacterial* proteins were identified by bidirectional best BLASTP hits (Altschul et al. 1997).

**FIGURE 2:**
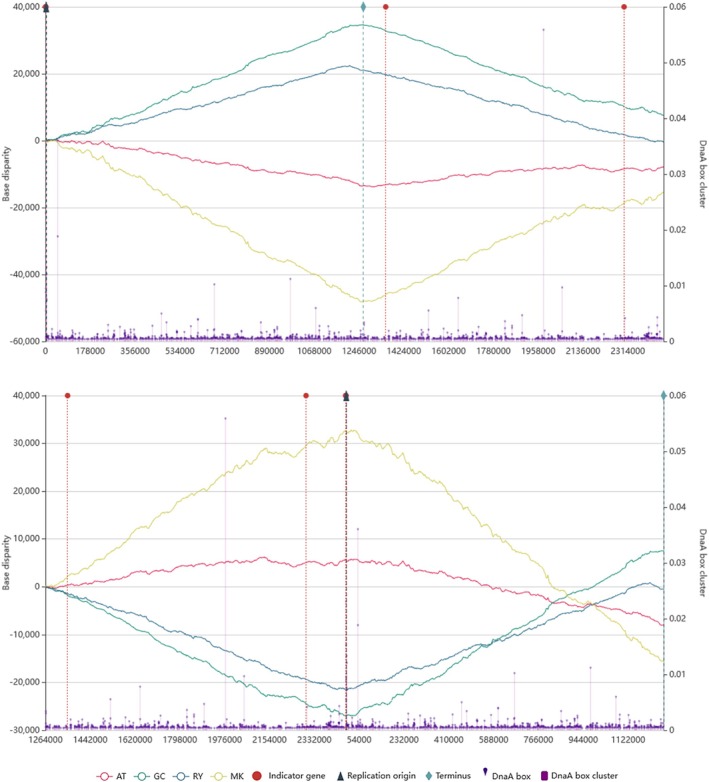
*Z*‐curves figures: native sequence (top) and rotated at the maximum of the GC disparity (bottom). The red, green, blue and yellow lines indicate AT, GC, RY and MK disparity. The purple marks indicate the dnaA box location. The red and blue dotted line indicate the locations of indicator genes and replication origins, respectively. Graphs were compiled via Ori‐Finder 2022 (tubic.tju.edu.cn).

Two proteins were considered orthologs if reciprocal BLASTP hits showed ≥ 30% identity and ≥ 50% query coverage, and each was the best hit in the other genome. Genes with orthologs in all selected corynebacterial genomes were assigned to the core genome. The following five genomes were used: *C. kroppenstedtii* subsp. *demodicis* (this study), *C. casei* UCMA 3821, *C. diphtheriae* 31A, *C. glutamicum* ATCC 13032, and *C. striatum* BM4687, all retrieved from the MicroScope Prokaryotic Genome Database (PkGDB; The NCBI accession numbers listed in Table S1). Genes were classified as singletons if no BLAST hit met the above criteria in any other genome (i.e., no reciprocal BLASTP hit with ≥ 30% identity and ≥ 50% query coverage in any of the other four reference genomes; five genomes total were included in this analysis). The origin of replication was identified by Ori‐Finder 2022 (tubic.tju.edu.cn) (Figure 2) (Dong et al. 2022).

The annotated genomic sequence of *C. kroppenstedtii* subsp. *demodicis* was submitted to the ENA/EMBL (European Nucleotide Archive, accession number PRJEB60059, BioProject PRJNA936850, BioSample SAMN33379427), GenBank (NCBI), and DDBJ databanks.

#### 2.5.1 Comparative Genomics

Functional classification of genes was performed using eggNOG v4.5.1 and eggNOG‐mapper v1.0.3 (Huerta‐Cepas et al. 2016). The genome was compared to the minimal gene set from Gil et al., which includes conserved housekeeping genes for basic metabolism and macromolecular biosynthesis, many of them essential (Gil et al. 2004).

**FIGURE 3:**
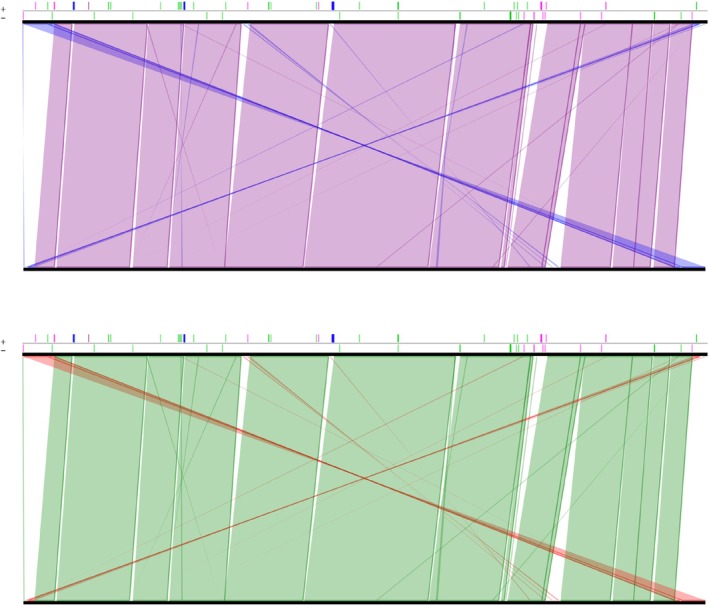
Global comparison based on conserved synteny results between *C. kroppenstedtii* subsp. *demodicis* (top) and *C. kroppenstedii* DSM 44385 (bottom) depicting an overview of the conservation of synteny groups: Inversions around the origin of replication (in red) (top) and strand conservations (in purple) and strand Inversions (in blue) (bottom). Transposases/insertion sequences are shown in pink, rRNA in blue and tRNA in green. Graphs were compiled via MicroScope (mage.genoscope.cns.fr).

Conserved synteny between *C. kroppenstedtii* subsp. *demodicis* and the reference genome was visualized using LinePlot with a synton size ≥ 3 genes (Figure 3).

**FIGURE 4:**
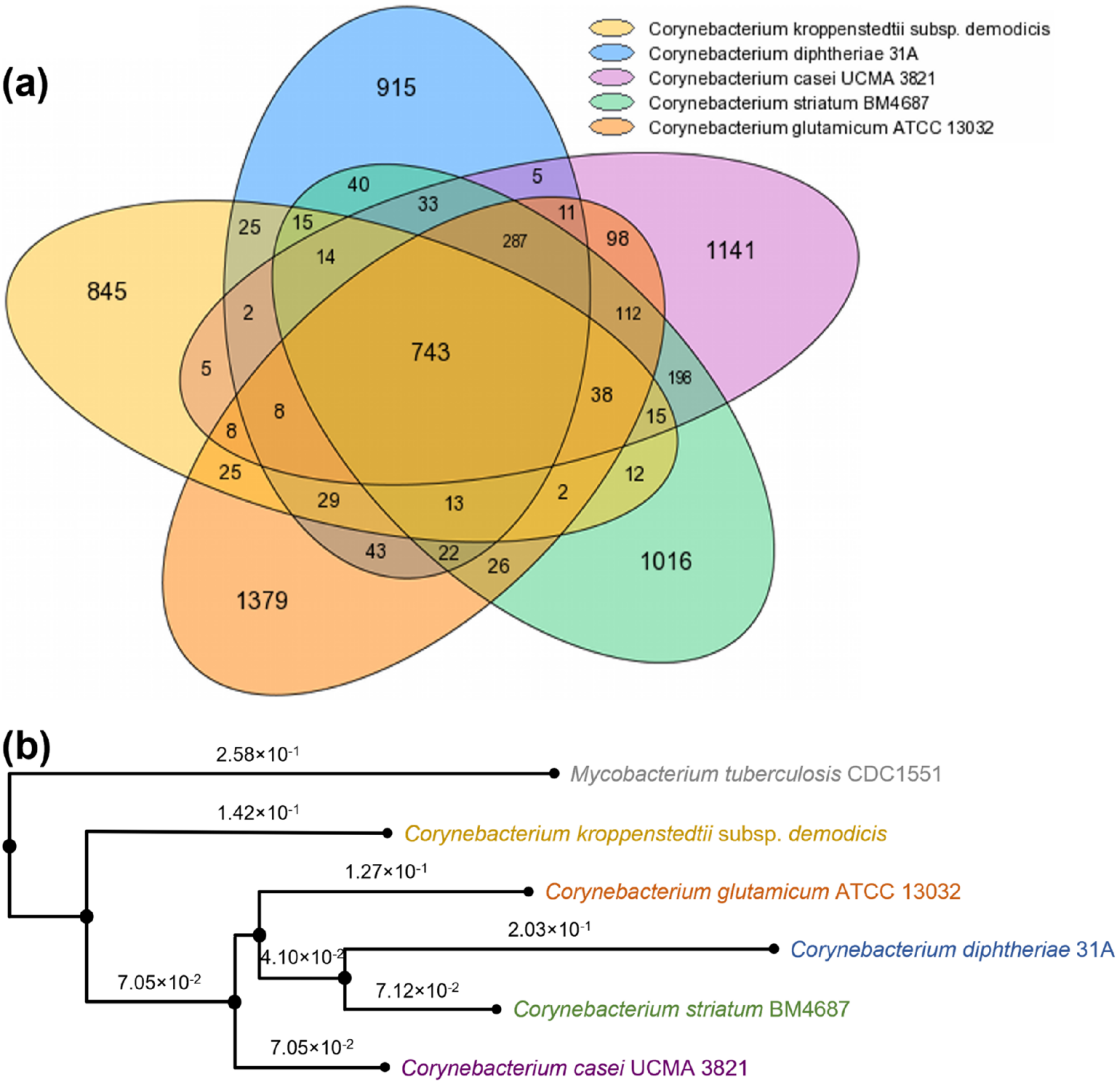
(a) Venn diagram of the pan‐, core‐ and variable genome of *C. kroppenstedii subsp. demodicis* (yellow), *C. diphtheriae* (blue), *C. casei* (purple), *C. striatum* (green) and *C. glutamicum* (orange). Depicted numbers are the numbers of gene families shared or not shared, respectively. (b) Genomic similarity estimated using Mash with clustering annotations. This distance D is correlated to the ANI like: D ≈ 1 − ANI. This clustering has been computed from all‐pairs distances ≤ 0.06 (≈94% ANI). *Mycobacterium tuberculosis* was used as outgroup.

The pan‐genome and its components (core genome, variable genome) were analyzed using MicroScope gene families (MICFAM) computed with SiLiX (Vallenet et al. 2020). Using one representative per *Corynebacterium* species from the MicroScope Prokaryotic Genome Database (PkGDB)— *C. casei* UCMA 3821, *C. diphtheriae* 31A, *C. glutamicum* ATCC 13032, *C. striatum* BM4687—thresholds of 50% amino acid identity and 80% alignment coverage were applied (Figure 4a).

Antibiotic resistance was predicted using the Comprehensive Antibiotic Resistance Database (CARD) and compared to the previously described experimental resistance profile (Clanner‐Engelshofen et al. 2020a). Proposed genes were manually screened for putative symbiotic roles using the MicroScope virulence factor database (VFDB) and VirulenceFinder data.

Genomic similarity was estimated with Mash, which computes the distance D between two genomes, where D is correlated with average nucleotide identity (ANI; D ≈ 1 − ANI). The resulting tree displays clustering (computed by Louvain community detection) based on all‐pairs distances ≤ 0.06 (≈94% ANI), corresponding to the ANI standard to define a species group (Figure 4b).

#### 2.5.2 Phylogenetic Analysis

Phylogenetic relationships were determined using the Type (Strain) Genome Server (TYGS, https://tygs.dsmz.de). Pairwise genomic comparisons were conducted using the Genome BLAST Distance Phylogeny (GBDP) approach with default parameters. Digital DNA–DNA hybridization (dDDH) values and confidence intervals were calculated with 100 distance replicates. A balanced minimum evolution tree with branch support was inferred using FastME 2.1.6.1, with branch support calculated from 100 pseudo‐bootstrap replicates.

**FIGURE 5:**
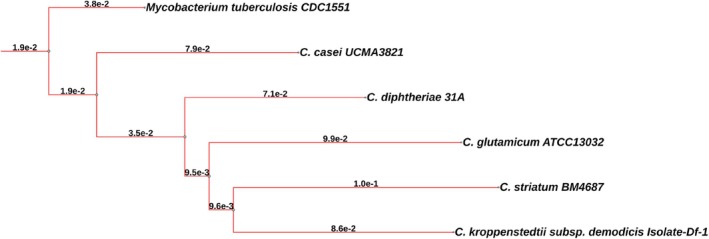
Phylogenetic tree of *Corynebacterium* species based on whole‐genome GBDP distances. *Mycobacterium tuberculosis* CDC1551 was used as an outgroup to root the tree. *C. kroppenstedtii* subsp. *demodicis* clusters appropriately within the *Corynebacterium* genus, showing its relationships to other corynebacterial species. Branch lengths represent evolutionary distances, with shorter branches indicating greater genomic similarity.

The resulting phylogenetic tree from TYGS was imported into the Interactive Tree of Life (iTOL, https://itol.embl.de) platform for visualization and formatting. The tree was rooted using the designated outgroup taxon ( *Mycobacterium tuberculosis* ) to ensure proper phylogenetic orientation. Branch support values provided by TYGS were displayed on the corresponding nodes. For clarity and presentation, taxon labels were adjusted and the tree layout was optimized. The final tree was exported as a high‐resolution figure for publication (Figure 5).

#### 2.5.3 Metabolism

Metabolic pathways were analyzed and visualized using KEGG and BioCyc (Karp et al. 2019; Kanehisa and Goto 2000). Secondary metabolite gene clusters were analyzed using antiSMASH v5.0.0 (Blin et al. 2019).

#### 2.5.4 Analysis of Symbiotic Traits

The *D. folliculorum* secretome was predicted from its published transcriptome using SignalP (Hu et al. 2019; Almagro Armenteros et al. 2019). The secretome of *C. kroppenstedtii* subsp. *demodicis* was predicted with the same tool. The secretomes were analyzed individually and combined using KEGG BlastKOALA (Kanehisa et al. 2016). Results were examined via KEGG pathways and Brite hierarchy to investigate symbiotic traits (Kanehisa and Goto 2000).

### 2.6 Scanning Electron Microscopy

Samples were mounted on glass slides, cover‐slipped, and cryo‐fixed in liquid nitrogen. After cover slip removal, samples were fixed for 30 min with 2.5% glutaraldehyde in 75 mM cacodylate buffer containing 150 mM NaCl and 2 mM MgCl_2_ at pH 7.0. Three buffer washes (5, 15, 30 min) were followed by 30 min in 1% OsO_4_, a 10 min buffer wash, and three washes in double‐distilled water. Dehydration was carried out in a graded acetone series with 10 min incubation at each step. Samples were then rinsed in 100% acetone (7, 20, 30 min, and overnight), critical‐point‐dried, and sputter‐coated with platinum for 40 s. Scanning electron microscopy was performed at 2.0 kV using SE detection on a Zeiss Auriga Crossbeam Station (Carl Zeiss AG, Oberkochen, Germany).

### 2.7 Brightfield and Fluorescence Microscopy

Microscopy was performed using an inverted Axio Observer 7 at 100×–400× magnification with ZEN Imaging Software (Zeiss). Mites were observed under visible (VIS) or UV light using blue (DAPI; Filtersets 02 or 49, Zeiss) or green (GFP; Filterset 38, Zeiss) filters. Autofluorescence of mites and their endobacteria aided localization in the pore plug.

## 3 Results

### 3.1 Genome of *C. kroppenstedtii* subsp. *demodicis*

#### 3.1.1 General Genome Properties and Chromosomal Architecture

The genome of *C. kroppenstedtii* subsp. *demodicis* is 2,456,075 bp in size, comprising a single circular replicon consistent with the previously described *C. kroppenstedtii* DSM44385 reference genome structure. The GC content is 57.2%, just 0.3% lower than in the reference (Table 1). Assembly quality metrics confirm a high‐quality genome with CheckM2 completeness of 97.96% and contamination of only 0.01%. BUSCO analysis against the actinomycetota_odb12 dataset (238 universal orthologous genes) revealed 96.2% complete single‐copy genes, with 2.1% fragmented and 1.7% missing, indicating excellent assembly completeness suitable for reliable comparative genomic analyses (Table S2).

**TABLE 1:** Features of the *C. kroppenstedtii* subsp. *demodicis* genome.

Feature	Data obtained after sequencing, annotation and partial manual curation
Genome size [bp]	2,456,075
Mean G + C content	57.18%
Predicted coding sequences	2034
Coding density	87.02%
Average gene length [bp]	1056.47
Average length of intergenic regions [bp]	167.13
Ribosomal RNAs	9 (= 3 × (16S + 23S + 5S))
Transfer RNAs	45

#### 3.1.2 Genome Synteny and Chromosomal Rearrangements

Comparative synteny analysis between *C. kroppenstedtii* subsp. *demodicis* and the reference strain *C. kroppenstedtii* DSM44385 revealed largely conserved chromosomal organization with several notable synteny breakpoints (Figure 3).

#### 3.1.3 Protein Content

Automatic annotation and partial manual curation using the MicroScope system identified 45 tRNA genes and 2034 coding sequences (CDS), which were classified into COG categories (Tatusov et al. 2000). Average CDS and intergenic lengths are 1056 bp and 167 bp, respectively, resulting in a protein coding density of 87% (Table 1).

Core genome analysis of *C. kroppenstedtii* subsp. *demodicis* and four other *Corynebacterium* species ( *C. diphtheriae* , *C. casei* , *C. striatum* , *C. glutamicum* ) identified 743 gene families containing 4491 genes. The variable genome was reduced by 7.7%–14.7% (vs. *C. diphtheriae* and *C. glutamicum* , respectively), leading to *C. kroppenstedtii* subsp. *demodicis* having the lowest percentage of variable genes (55.9%) and the highest percentage of core genes (44.1%) (Figure 4a). The pan‐genome comprised 13,312 genes in 7125 families, and the variable genome comprised 8821 genes in 6382 families. These variable genome estimates are based on single reference strains per species. The inclusion of additional strains would likely increase the variable genome proportions for all species and may narrow the apparent difference between *C. kroppenstedtii* subsp. *demodicis* and its relatives.

#### 3.1.4 Phylogenetic Relationships and Genomic Distances

TYGS‐based phylogenetic analysis using GBDP distances confirmed the placement of *C. kroppenstedtii* subsp. *demodicis* within the *Corynebacterium* genus (Figure 5). The analysis revealed closest relationships with other human‐associated corynebacterial species, with clear separation from the mycobacterial outgroup (Table S4). The phylogenetic tree exhibited moderate branch support values, confirming relationships within the corynebacterial clade. While the pangenome analysis provided insights into gene family distributions, phylogenetic analysis based on whole‐genome sequences revealed detailed evolutionary relationships among corynebacterial lineages.

### 3.2 Predicted Proteome of *C. kroppenstedtii* subsp. *demodicis*

#### 3.2.1 Primary Metabolism

KEGG pathway analysis revealed numerous incomplete metabolic pathways, particularly in carbohydrate biosynthesis and cofactor synthesis, reflecting the organism's adaptation to a symbiotic lifestyle (Table S3). Compared to the minimal gene set of Gil et al. (2004), *C. kroppenstedtii* subsp. *demodicis* lacks several essential genes. Instead of asparaginyl‐tRNA synthase, it employs a promiscuous aspartyl‐tRNA synthetase that also recognizes asparaginyl‐tRNA. Glutaminyl‐tRNA synthase is missing. Glutaminyl‐tRNA is produced by glutamyl‐tRNA amidotransferase from glutamyl‐tRNA.

Glycero(phospho)lipid metabolism lacks phosphatidylserine decarboxylase and synthase, resulting in phosphatidylinositol, phosphatidylglycerol, cardiolipin, and acylphosphatidylglycerol as the main phospholipids, with phosphatidylethanolamine absent, as seen in other Corynebacteria (Yague et al. 2003; Valenzuela Baez 2013). Additionally, the sn‐glycerol‐3‐phosphate acyltransferase gene *plsB* is missing, suggesting that 1‐acyl‐glycerol‐3‐phosphate is synthesized by the two‐enzyme system PlsX/Y (Gully and Bouveret 2006).

In nicotinamide adenine dinucleotide (NAD+) biosynthesis, the organism lacks N‐ribosyl nicotinamide kinase, strongly suggesting synthesis via phosphoribosyl diphosphate.

Compared to the other four Corynebacteria analyzed, the genome of *C. kroppenstedtii* subsp. *demodicis* shows additional unique features. It lacks phosphoenolpyruvate carboxylase used for gluconeogenesis, as well as alcohol and acetaldehyde dehydrogenases, preventing pyruvate fermentation to ethanol but allowing conversion to lactate or acetate. Multiple carbohydrate and cofactor biosynthesis pathways are incomplete due to missing enzymes (dTDP‐4‐dehydrorhamnose 3,5‐epimerase for dTDP‐L‐rhamnose, mannose‐1‐phosphate guanylyltransferase for GDP‐mannose, maltodextrin and glycogen phosphorylase for glycogen degradation; molybdenum cofactor guanylyltransferase for guanylyl molybdenum cofactor, 2‐dehydropantoate 2‐reductase for phosphopantothenate).

A striking metabolic feature is the absence of genes for fatty acid synthase type I (FAS I), which initiates fatty acid biosynthesis and explains the obligate lipophilism.

Xanthine dehydrogenase is missing, so endogenous xanthine is likely salvaged for purine nucleotide synthesis. Exogenous urate/uric acid is metabolized via a uricolytic pathway involving allantoicase, allantoinase, and urease. In nitrogen metabolism, the endobacteria lack ammonia monooxygenase but possess the ammonium transporter gene *amtB*, indicating that under low oxygen and high UV exposure, excess ammonium may be removed directly without conversion to nitrite/nitrate (see below).

#### 3.2.2 Secondary Metabolism: General Features

Contrary to previous reports (Collins et al. 1998), *C. kroppenstedtii* subsp. *demodicis* is genetically equipped to synthesize corynemycolic acids, a feature recently confirmed phenotypically via mass spectrometry by our group (Clanner‐Engelshofen et al. 2020a).

For iron acquisition, multiple ABC transporters for siderophores and hemophores are encoded, primarily for ferric enterobactin. AntiSMASH and KEGG analyses revealed no hitherto known siderophore pathway. However, genes for 2,3‐dihydroxybenzoate biosynthesis (which may explain the distinctive fluorescent phenotype, authors' observation) suggest a catecholate siderophore, such as enterobactin or bacillibactin, but not corynebactin as in *C. diphtheriae* (Dertz et al. 2006; Zajdowicz et al. 2012). A non‐ribosomal peptide synthase cluster (bp 2,176,100–2,228,240) suggests a peptide monomer comprising one unspecified amino acid, glutamine, and dihydroxybenzoate. Additionally, the genome encodes L‐lysine N6‐monooxygenase (*mbtG*), supporting the possibility of a heterobactin‐type siderophore.

### 3.3 Endosymbiotic Traits

#### 3.3.1 Secondary Metabolism: Putatively Aiding Symbiosis

**FIGURE 6:**
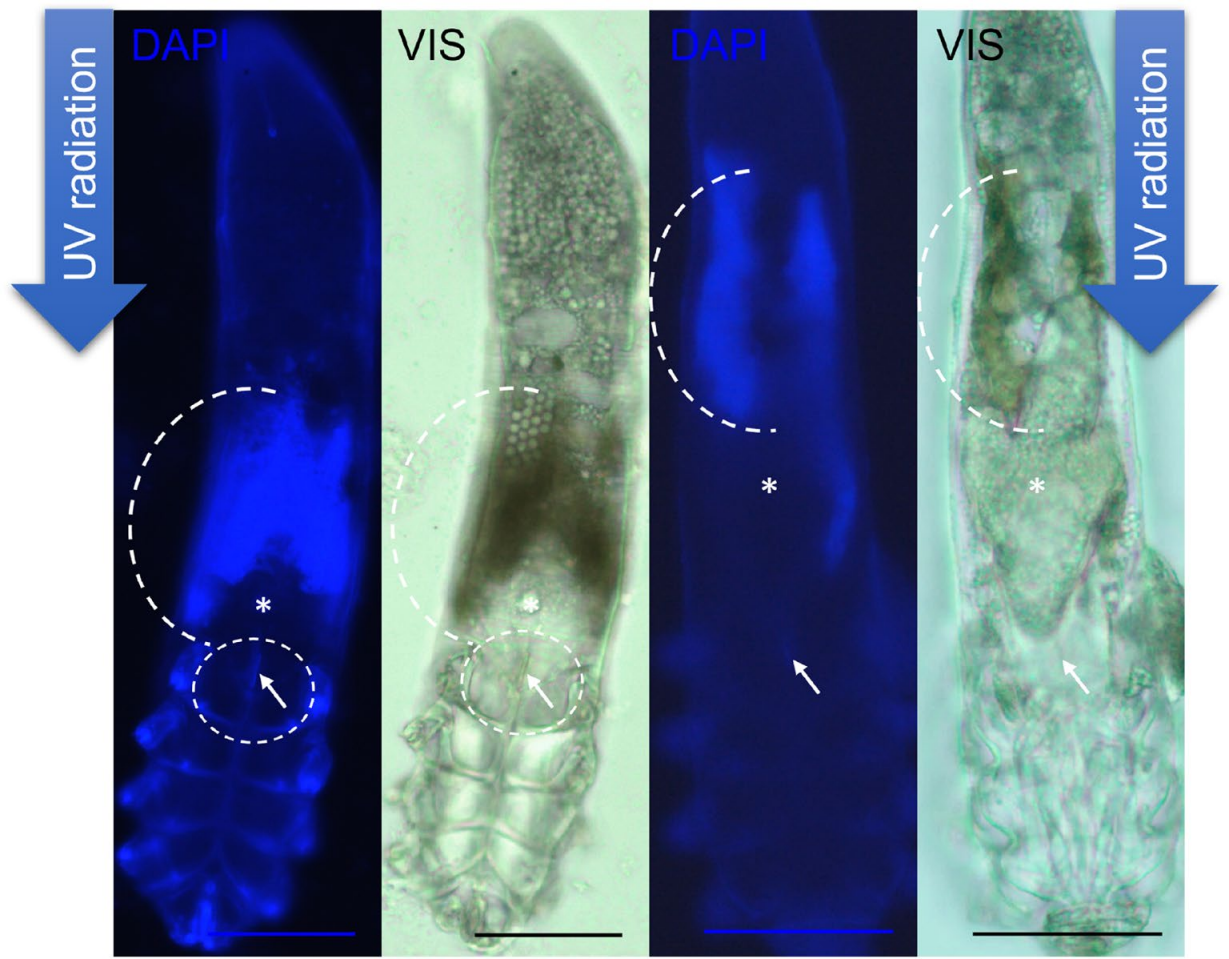
Left: DAPI and VIS image of a non‐oviparous, female mite. Right: DAPI and VIS image of an oviparous, female mite. The localization of the endobacteria (brownish colour in VIS images) in the midgut is marked by a dashed half‐circle, the synganglion (primitive “brain”) by a dashed full circle (left, not visible in the right image). The vaginal opening is marked by an arrow, the uterus by an asterisk, containing an arrowhead‐shaped egg in the right image. The direction of UV radiation exposure is indicated by arrows (caudal to cranial). Scale bars = 50 μm.

Unlike many other Corynebacteria, *C. kroppenstedtii* subsp. *demodicis* has a complete mevalonate pathway for the biosynthesis of β‐carotene, a molecule with UV‐protective and antioxidant properties. Terpenoid backbone biosynthesis is not supported via the methylerythritol phosphate pathway due to missing enzymes: 1‐deoxy‐D‐xylulose‐5‐phosphate synthase, 1‐deoxy‐D‐xylulose‐5‐phosphate reductoisomerase, 2‐C‐methyl‐D‐erythritol 4‐phosphate cytidylyltransferase, 2‐C‐methyl‐D‐erythritol 2,4‐cyclodiphosphate synthase, (E)‐4‐hydroxy‐3‐methylbut‐2‐enyl‐diphosphate synthase, and 4‐hydroxy‐3‐methylbut‐2‐enyl diphosphate reductase. All other *Corynebacteria* analyzed use the methylerythritol phosphate pathway and have an incomplete mevalonate pathway. The genome encodes a complete β‐carotene biosynthetic pathway, and endobacteria are localized caudal to the UV‐sensitive synganglion and uterus (Figure 6). β‐carotene is known to exhibit UV‐absorbing properties. In addition, the genome encodes multiple systems associated with oxidative and nitrosative stress responses. These include manganese‐dependent superoxide dismutase (SodA), catalase (KatA), a manganese ABC transporter (SitB), and several peroxidases involved in detoxification of H_2_O_2_ and other reactive oxygen species. Oxidatively damaged cellular components may be repaired or degraded by DNA repair protein RecN, peptide‐methionine (S)‐S‐oxide reductase, and heat shock protein ClpB. Genes encoding nitrite reductase and cytochrome c oxidase are present, consistent with potential detoxification of reactive nitrogen species such as nitrite and nitric oxide (Meng et al. 2018). Additionally, the genome contains a complete *mshABCD* cluster for mycothiol biosynthesis, a glutathione‐like scavenger of radicals, RNS, ROS and alkylating agents, and mycothiol S‐conjugate amidase for recycling.

#### 3.3.2 Microscopy

The mites host endobacteria in their midgut, as previously described (Clanner‐Engelshofen et al. 2020a). Detailed inspection reveals that key organs: the synganglion (nuclei‐rich, primitive “brain”) and the uterus (harbouring offspring) are spatially associated with dense accumulations of endobacteria when residing in the follicle. These bacterial clusters exhibit a distinct brownish pigmentation in bright‐field microscopy, consistent with carotenoid production (see Figure 6).

#### 3.3.3 Secretome Putatively Involved in Endosymbiosis

**FIGURE 7:**
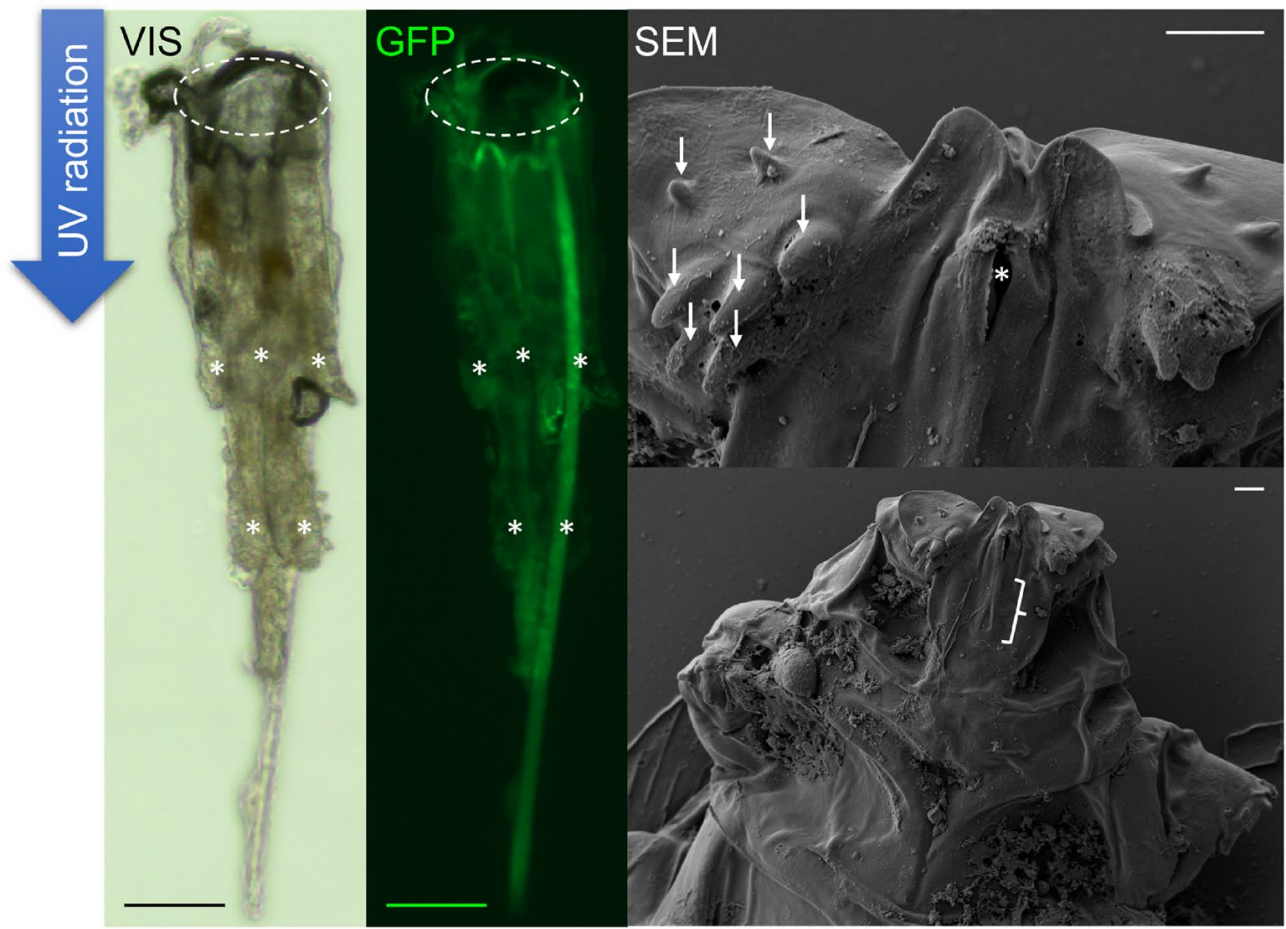
VIS and GFP image of an extracted pore plug containing 5 mites (podosoma marked by asterisks) and a vellus hair. The opening of the pore is localized in the upper part of the image (dashed circle). The direction of UV radiation exposure is indicated by an arrow. The mites are situated head‐downwards and tail toward the follicular opening, with their ventral side toward the follicular epithelium. Scale bars = 100 μm SEM image of the mite's gnathosoma (upper: Detail, lower: Overview). Asterisk: oral opening containing the stylet. Arrows: palpal claws. Brace: hypostoma. Scale bars = 2 μm.

Microscopic analysis (including authors' observations) shows that intrafollicular mites are oriented with their gnathosoma toward the stratum corneum (Figure 7 VIS/GFP). The stratum corneum is around 10 μm thick, while palpal claws measure approximately 1–1.5 μm (Figure 7 SEM). Mites seem to avoid deeper, non‐keratinized epidermal layers and instead graze on the most superficial keratin layers—supposedly only when the follicle is not pathologically overpopulated by mites, as in immunodeficient hosts. The resulting chyme, partially digested pre‐orally (as the mite's oesophagus is just about 1 μm in diameter), is further processed in the midgut.

Here, an endoglycoceramidase hydrolyzes the β‐glycosidic linkage between oligosaccharides and ceramides. The endobacteria also secrete phospholipase C, which hydrolyzes phosphatidylcholine, phosphatidylethanolamine and sphingomyelin found in human cell membranes, along with glycerophosphoryl diester phosphodiesterase. They subsequently encode ethanolamine ammonia‐lyase to degrade ethanolamine (a component of phosphatidylethanolamine) for nitrogen utilization. Host phosphate‐hydrolyzing enzymes (alkaline phosphatase A and B) are complemented by bacterial alkaline phosphatase D. The mite's triacylglycerol lipase is supported by a bacterial homologue. To degrade complex sugars, the endobacteria also secrete β‐glucosidase and exo‐α‐sialidase (NanI, previously not amplifiable by polymerase‐chain reaction) (Clanner‐Engelshofen et al. 2020a). Exogenous glutathione and peptides or proteins in the human epidermis are likely degraded by gamma‐glutamyltranspeptidase/glutathione hydrolase and broad‐specificity aminopeptidase N.

### 3.4 Resistome

The previously described antibiotic susceptibility profile (Clanner‐Engelshofen et al. 2020a) was analyzed genetically. The endobacteria encode the 23S rRNA methyltransferase Erm, which methylates rRNA and confers resistance to lincomycin and clindamycin. Erm‐methylation may also explain intermediate susceptibility to macrolides such as erythromycin.

Phenotypic fosfomycin resistance may result from a point mutation in UDP‐N‐acetylglucosamine 1‐carboxyvinyltransferase MurA (Asp50Glu), previously described in *Enterococcus faecium* (Xin et al. 2022).

## 4 Discussion

This study provides compelling insights into the adaptive strategies of *Corynebacterium kroppenstedtii* subsp. *demodicis*, emphasizing its distinctive features within a symbiotic context. Compared to closely related strains, this endobacterium exhibits unique biochemical and structural adaptations crucial for survival and functional integration within the mite midgut. The GC content of the isolate is 57.3 mol%, while that of *C. kroppenstedtii* DSM 44385 is 62.0 mol%, as determined by the DSMZ. This implies that the genetic material is largely conserved between the two strains, with slight variation in GC base composition.

The synteny breakpoints indicate localized genomic rearrangements that have occurred during the evolutionary divergence of subsp. *demodicis* from the reference strain. The presence of chromosomal inversions and rearrangements suggests ongoing genome plasticity within the *C. kroppenstedtii* lineage, consistent with previous observations of moderate genome reorganization in some corynebacterial species (Tauch et al. 2008). Despite these local rearrangements, the overall gene content and organization remain highly conserved between the two strains, supporting their close phylogenetic relationship while highlighting strain‐specific evolutionary adaptations.

The TYGS phylogenetic analysis based on whole‐genome sequences corroborates these evolutionary relationships, positioning *C. kroppenstedtii* subsp. *demodicis* closest to *C. striatum* , a human skin‐associated opportunistic pathogen (Table S4). This phylogenetic proximity suggests convergent evolution toward human‐associated lifestyles despite distinct ecological niches. The moderate branch support values reflect the relatively recent diversification within the *Corynebacterium* genus, consistent with rapid adaptation to diverse host environments. A key adaptation of the isolate is the presence of corynemycolic acids—structural components characteristic of the CMNR group that enhance resistance to chemical and antibiotic stress (Burkovski 2018). This mycomembrane protects against the acidic midgut environment, where the pH ranges from 4.5 to 7 (Spickett 1961). Similar mechanisms in *Mycobacterium tuberculosis* help maintain intracellular pH homeostasis at 4.5 (Clanner‐Engelshofen et al. 2020a). As mites digest keratin using acidic cysteine proteases like Der f1 and Der p1 (Byrd et al. 2018), corynemycolic acids likely prevent proton influx, ensuring bacterial survival. These features underscore the bacterium's host‐specific niche adaptation.

The niche of *C. kroppenstedtii* subsp. *demodicis* in the mite's lipid‐rich midgut enables reliance on host‐derived fatty acids. This metabolic specialization is evident in the lack of endogenous fatty acid synthesis (FAS I). Functionally, this adaptation complements host lipid and carbohydrate metabolism, but less so protein metabolism, given the extensive proteolytic capacity of the mite's secretome, which contains 26 proteases. This mutualism highlights the bacterium's role in nutrient processing rather than protein degradation.

Compared to canonical insect endosymbionts (e.g., *Buchnera aphidicola* : ~650 kb (Shigenobu et al. 2000); *Blochmannia floridanus*: ~710 kb (Gil et al. 2003)), the genome of *C. kroppenstedtii* subsp. *demodicis* (2.46 Mb) is relatively large, suggesting an earlier evolutionary stage of endosymbiotic genome reduction. The retention of a substantial accessory genome (55.9% variable genes, see Table 1) is consistent with a facultative‐to‐obligate transition still under way, as has been proposed for some horizontally transmitted endosymbionts (Clayton et al. 2012).

A distinctive feature of *C. kroppenstedtii* subsp. *demodicis* is its complete mevalonate pathway for β‐carotene biosynthesis. We hypothesize that the endobacterium contributes to UV protection via β‐carotene production, based on (i) the presence of a complete biosynthetic pathway, (ii) its anatomical localization caudal to UV‐sensitive host structures such as the synganglion and uterus, and (iii) the established UV‐absorbing properties of carotenoids. However, direct in vivo evidence for this role is currently lacking. Unlike other Corynebacteria utilizing the methylerythritol phosphate pathway, this bacterium's β‐carotene production is consistent with UV‐protective and antioxidant properties. This is vital in mites, as microscopy shows endobacteria localized caudal to the synganglion and the uterus—organs susceptible to UV damage. These endobacteria exhibit a distinct brownish pigmentation in bright‐field microscopy, consistent with carotenoid accumulation. Since light enters the follicle caudally, cranial organs are protected. β‐carotene may shield these structures, potentially supporting neurological function and reproductive success. In addition to potential photoprotection, the encoded metabolic repertoire suggests a broader role in mitigating oxidative and nitrosative stress. Beyond passive UV protection, the bacterium actively mitigates oxidative stress through antioxidant mechanisms. Manganese‐dependent superoxide dismutase (SodA), catalase (KatA), and manganese ABC transporter (SitB) neutralize hydrogen peroxide and other ROS. Proteins such as DNA repair protein RecN and peptide‐methionine (S)‐S‐oxide reductase protect cellular macromolecules from oxidative damage. Nitrosative stress from UV‐induced nitrite and nitric oxide (NO) is quenched by nitrite reductase and cytochrome‐c oxidase. Additionally, the mshABCD gene cluster for mycothiol biosynthesis detoxifies radicals, RNS, and ROS, highlighting a sophisticated defense system.

Endobacteria are thus strategically localized to protect vital mite organs. The synganglion, rich in neural nuclei, is vulnerable to oxidative stress that may impair neurological function. Similarly, the uterus, essential for offspring development, requires protection from UV‐induced damage. β‐carotene and antioxidant systems ensure the integrity of these vital organs, supporting the mite's survival and reproductive success. These findings reveal an intricate co‐evolution between *C. kroppenstedtii* subsp. *demodicis* and its mite host, highlighting biochemical traits for survival in acidic and UV‐exposed environments.

### 4.1 Implications for Human Skin Ecology and Dermatology

*Demodex* mites and their endobacteria are increasingly recognized as relevant actors in human skin ecology. Elevated *Demodex* density has been associated with conditions such as rosacea, ocular demodicosis, and perioral dermatitis, and disruption of the mite–bacterium symbiosis may contribute to disease pathogenesis (Clanner‐Engelshofen et al. 2020a). The metabolic capabilities identified in this study, including: lipid‐processing enzymes, antioxidant systems, and the putative UV‐protective β‐carotene biosynthesis may influence the mite's colonization fitness and host immune response in dysbiotic skin states. Future work should investigate whether the endobacterium's predicted functions differ between healthy and diseased hosts, and whether targeting the symbiosis (e.g., in vitro inhibition of the mevalonate pathway) represents a viable therapeutic strategy in *Demodex*‐associated dermatoses.

## Author Contributions

**L. E. French:** resources, supervision, project administration, writing – review and editing. **M. Flaig:** project administration, resources, supervision. **S. Walch:** data curation, validation, writing – review and editing. **T. Steegmüller:** conceptualization, investigation, funding acquisition, writing – original draft, writing – review and editing, visualization, validation, methodology, software, formal analysis, data curation. **S. Gschwendtner:** validation, data curation, resources, writing – review and editing. **B. M. Clanner‐Engelshofen:** writing – review and editing, resources, supervision, formal analysis, project administration. **A. Klingl:** resources, data curation, writing – review and editing.

## Funding

This work was supported by Medical & Clinician Scientist Program (MCSP) at LMU Munich.

## Ethics Statement

The use of anonymized patient material was approved by the local ethics committee (project 17‐450 UE and 18‐671 UE).

## Conflicts of Interest

The authors declare no conflicts of interest.

## Supporting information


**Table S1:** GenBank accession numbers of all genome sequences used for comparative genomic analyses.
**Table S2:** Assembly quality assessment of *C. kroppenstedtii* subsp. *demodicis* genome. Assembly quality was evaluated using CheckM2 v1.0.1 and BUSCO v5.8.0 with the actinomycetota_odb12 lineage dataset (238 BUSCOs). CheckM2 estimates are based on conserved marker gene analysis, while BUSCO assesses completeness using universal single‐copy orthologous genes.
**Table S3:** KEGG pathway analysis showing incomplete and complete metabolic pathways. Pathways assessed using KEGG BlastKOALA and antiSMASH v5.0.0.
**Table S4:** Digital DNA‐DNA hybridization (dDDH) distances: Pairwise genomic distances calculated using TYGS platform with GBDP algorithm. Values shown with 95% confidence intervals based on 100 distance replicates. GC content differences indicate genomic compositional divergence between species.

## Data Availability

The data that support the findings of this study are available on request from the corresponding author. Some data are not publicly available due to privacy or ethical restrictions. The data regarding the genome sequencing and annotations are openly available in ENA/EMBL (European Nucleotide Archive), GenBank (NCBI), and DDBJ databanks.
