# Supplementary material for: Third Generation Genome Sequencing of the Endobacterium Corynebacterium kroppenstedtii subsp. demodicis Reveals Details of Its Microbe‐Host‐Interaction With the Most Complex Human Commensal, Demodex folliculorum
Source: Environ Microbiol Rep. 2026 Jun 8;18(3):e70374. doi: 10.1111/1758-2229.70374 (PMC13244258; doi:10.1111/1758-2229.70374)
Supplement: Supplementary file 1 — Table S1: GenBank accession numbers of all genome sequences used for comparative genomic analyses. Table S2: Assembly quality assessment of C. kroppenstedtii subsp. demodicis genome. Assembly quality was evaluated using CheckM2 v1.0.1 and BUSCO v5.8.0 with the actinomycetota_odb12 lineage dataset (238 BUSCOs). CheckM2 estimates are based on conserved marker gene analysis, while BUSCO assesses completeness using universal single‐copy orthologous genes. Table S3: KEGG pathway analysis showing incomplete and complete metabolic pathways. Pathways assessed using KEGG BlastKOALA and antiSMASH v5.0.0. Table S4: Digital DNA‐DNA hybridization (dDDH) distances: Pairwise genomic distances calculated using TYGS platform with GBDP algorithm. Values shown with 95% confidence intervals based on 100 distance replicates. GC content differences indicate genomic compositional divergence between species. [file EMI4-18-e70374-s001.docx]

**Supplementary files for review**

**Table S 1** Accession number of each species

| **Species** | **Accession number** |
| --- | --- |
| C. kroppenstedtii subsp. demodicis | GCF_045277265 |
| C. glutamicum ATCC 13032 | GCF_000011325 |
| C. diphtheriae 31A | GCF_000241875 |
| C. striatum BM4687 | GCA_001261935 |
| C. casei UCMA 3821 | GCF_000234765 |
| C. kroppenstedtii DSM 44385 | GCF_000023145 |
| Mycobacterium tuberculosis CDC1551 | GCF_000008585 |

**Table S 2** Assembly quality assessment of C. kroppenstedtii subsp. demodicis genome. Assembly quality was evaluated using CheckM2 v1.0.1 and BUSCO v5.8.0 with the actinomycetota_odb12 lineage dataset (238 BUSCOs). CheckM2 estimates are based on conserved marker gene analysis, while BUSCO assesses completeness using universal single-copy orthologous genes.

| **Metric** | **Value** |
| --- | --- |
| Assembly size (bp) | 2,456,075 |
| GC Content (%) | 57.2 |
| CheckM2 completeness (%) | 97.96 |
| CheckM2 contamination (%) | 0.01 |
| CheckM2 coding density (%) | 88.2 |
| CheckM2 total coding sequences | 2150 |
| BUSCO lineage dataset | actinomycetota_odb12 |
| Total BUSCO groups searched | 238 |
| BUSCO complete single-copy (%) | 96.2 |
| BUSCO complete duplicated (%) | 0 |
| BUSCO fragmented (%) | 2.1 |
| BUSCO missing (%) | 1.7 |
| Sequencing platform | PacBio Sequel |
| Mean read coverage (×) | 30 |

**Table S 3** KEGG pathway analysis showing incomplete and complete metabolic pathways. Pathways assessed using KEGG BlastKOALA and antiSMASH v5.0.0.

| **Pathway Name** | **KEGG ID** | **Status** | **Missing Enzymes (EC/KO numbers)** | **Functional Impact** |
| --- | --- | --- | --- | --- |
| Fatty acid biosynthesis | ko00061 | Incomplete | Fatty acid synthase type I (FAS I) system | Explains obligate lipophilism; must obtain lipids from host |
| dTDP-L-rhamnose biosynthesis | ko00521 | Incomplete | dTDP-4-dehydrorhamnose 3,5-epimerase (rfbC) | Cannot synthesize this cell wall sugar component |
| GDP-mannose biosynthesis | ko00051 | Incomplete | mannose-1-phosphate guanylyltransferase (manC) | Limited carbohydrate processing capacity |
| Glycogen degradation | ko00500 | Incomplete | maltodextrin/glycogen phosphorylase (glgP) | Cannot break down stored carbohydrates |
| Ethanol fermentation | ko00010 | Incomplete | alcohol dehydrogenase (adhE), acetaldehyde dehydrogenase | Pyruvate metabolized to lactate/acetate only |
| Gluconeogenesis | ko00020 | Incomplete | phosphoenolpyruvate carboxylase (ppc) | Reduced glucose synthesis from non-carbohydrate sources |
| Methylerythritol phosphate pathway | ko00900 | Incomplete | 2-C-methyl-D-erythritol 4-phosphate cytidylyltransferase, others | Uses alternative mevalonate pathway for terpenoids |
| Molybdenum cofactor biosynthesis | ko00790 | Incomplete | molybdenum cofactor guanylyltransferase (mobA) | Limited Mo-dependent enzyme function |
| Phosphopantothenate biosynthesis | ko00770 | Incomplete | 2-dehydropantoate 2-reductase (panE) | Incomplete CoA precursor synthesis |
| NAD+ biosynthesis | ko00760 | Incomplete | N-ribosyl nicotinamide kinase (pnuC) | Uses alternative PRPP-dependent pathway |
| Phosphatidylethanolamine biosynthesis | ko00564 | Incomplete | phosphatidylserine decarboxylase, phosphatidylserine synthase | Limited membrane phospholipid diversity |
| 1-acyl-glycerol-3-phosphate biosynthesis | ko00561 | Incomplete | sn-glycerol-3-phosphate acyltransferase (plsB) | Uses PlsX/Y two-enzyme system instead |
| Purine salvage | ko00230 | Incomplete | xanthin dehydrogenase (xdhA) | Relies on exogenous purine sources |
| Ammonia oxidation | ko00910 | Incomplete | ammonia monooxygenase (amoA) | Direct ammonium removal via amtB transporter |
| β-carotene biosynthesis | ko00906 | Complete | None | UV protection via complete mevalonate pathway |
| Mycothiol biosynthesis | ko00920 | Complete | mshA-D cluster all present | ROS/RNS detoxification system |
| Uricolytic pathway | ko00230 | Complete | allantoicase, allantoinase, urease present | Urate/uric acid metabolism and solubilization |
| Iron acquisition | ko02010 | Complete | ferric enterobactin ABC transporter, siderophore genes | Multiple iron uptake mechanisms |
| Antioxidant defense | ko00480 | Complete | SodA, KatA, RecN, ClpB, MsrA present | Comprehensive oxidative stress protection |
| Antibiotic resistance | ko01501 | Complete | Erm methyltransferase, TetW, MurA variants | Resistance to lincomycin, clindamycin, fosfomycin |

Table S 4 digital DNA-DNA hybridization (dDDH) distances: Pairwise genomic distances calculated using TYGS platform with GBDP algorithm. Values shown with 95% confidence intervals based on 100 distance replicates. GC content differences indicate genomic compositional divergence between species.

| **Species 1** | **Species 2** | **dDDH (d4, %)** | **C.I. (95%)** | **GC diff (%)** |
| --- | --- | --- | --- | --- |
| *C. kroppenstedtii subsp. demodicis* | *C. striatum* | 26.1 | [23.7-28.5] | 2.19 |
| *C. kroppenstedtii subsp. demodicis* | *C. glutamicum* | 26.0 | [23.6-28.5] | 3.37 |
| *C. kroppenstedtii subsp. demodicis* | *C. diphtheriae* | 23.5 | [21.2-26.0] | 3.56 |
| *C. kroppenstedtii subsp. demodicis* | *C. casei* | 22.5 | [20.2-24.9] | 1.84 |
| *C. kroppenstedtii subsp. demodicis* | *M. tuberculosis* | 23.1 | [20.9-25.6] | 8.43 |
| *C. striatum* | *C. diphtheriae* | 26.8 | [24.4-29.2] | 5.74 |
| *C. glutamicum* | *C. diphtheriae* | 24.1 | [21.8-26.5] | 0.19 |
| *C. casei* | *C. diphtheriae* | 23.3 | [21.0-25.8] | 1.72 |
| *C. casei* | *C. glutamicum* | 23.3 | [21.0-25.8] | 1.53 |
| *C. casei* | *C. striatum* | 21.1 | [18.9-23.6] | 4.02 |
| *C. diphtheriae* | *M. tuberculosis* | 29.6 | [27.2-32.1] | 11.99 |
| *C. glutamicum* | *M. tuberculosis* | 23.7 | [21.4-26.2] | 11.80 |
| *C. casei* | *M. tuberculosis* | 30.2 | [27.8-32.7] | 10.27 |
| *C. striatum* | *M. tuberculosis* | 18.1 | [16.0-20.5] | 6.25 |
